# Antibacterial and Sporicidal Activity Evaluation of Theaflavin-3,3′-digallate

**DOI:** 10.3390/ijms23042153

**Published:** 2022-02-15

**Authors:** Ayuni Yussof, Brian Cammalleri, Oluwanifemi Fayemiwo, Sabrina Lopez, Tinchun Chu

**Affiliations:** Department of Biological Sciences, Seton Hall University, South Orange, NJ 07079, USA; mohamesi@shu.edu (A.Y.); cammalbr@shu.edu (B.C.); fayemiol@shu.edu (O.F.); lopezsac@shu.edu (S.L.)

**Keywords:** antibacterial, sporicidal, anti-germination, binding analysis, natural product, black tea polyphenol, theaflavin

## Abstract

Theaflavin-3,3′-digallate (TFDG), a polyphenol derived from the leaves of *Camellia sinensis*, is known to have many health benefits. In this study, the antibacterial effect of TFDG against nine bacteria and the sporicidal activities on spore-forming *Bacillus* spp. have been investigated. Microplate assay, colony-forming unit, BacTiter-Glo^TM^, and Live/Dead Assays showed that 250 µg/mL TFDG was able to inhibit bacterial growth up to 99.97%, while 625 µg/mL TFDG was able to inhibit up to 99.92% of the spores from germinating after a one-hour treatment. Binding analysis revealed the favorable binding affinity of two germination-associated proteins, GPR and Lgt (GerF), to TFDG, ranging from −7.6 to −10.3 kcal/mol. Semi-quantitative RT-PCR showed that TFDG treatment lowered the expression of *gpr*, ranging from 0.20 to 0.39 compared to the control in both *Bacillus* spp. The results suggest that TFDG not only inhibits the growth of vegetative cells but also prevents the germination of bacterial spores. This report indicates that TFDG is a promising broad-spectrum antibacterial and anti-spore agent against Gram-positive, Gram-negative, acid-fast bacteria, and endospores. The potential anti-germination mechanism has also been elucidated.

## 1. Introduction

Many medical and scientific journal articles have documented the rising number of antibiotic-resistant bacteria and the multidrug resistance crisis linked to the overuse or abuse of antibiotics [1]. Vancomycin, for example, was first introduced to clinical practice in 1972, and unfortunately, vancomycin-resistant *S. aureus* (VRSA) was reported in 1979 [2]. In the United States, approximately 2.8 million people are infected with antibiotic-resistant bacteria yearly, and at least 35,000 die from the infection [3]. The problem of antibiotic resistance imposes a significant financial burden as evidenced by the number of methicillin-resistant *Staphylococcus aureus* (MRSA)-related issues that cost the US healthcare system around $3–4 billion annually [4]. 

The aromatic allure, taste, and health benefits of tea make it one of the most popular beverages worldwide [5]. Both black and green tea are derived from the leaves of *Camellia sinensis* but differ in the level of oxidation due to fermentation [5,6]. Black tea contains a lower level of catechins than green tea but makes up for it with a higher amount of theaflavin [6]. The major theaflavins present in black tea include theaflavin (TF), theaflavin-3-gallate (TF3G), theaflavin-3′-gallate (TF3′G), and theaflavin-3,3′-digallate (TFDG) [6]. 

Theaflavins (TFs) are the major polyphenols in black tea, showing great potential as an antimicrobial agent. A previous study demonstrated that 1 g of theaflavin mixture extract could contain up to 32.80% of TFDG [7]. As for cellular toxicity, theaflavin has little to no effect on human lung fibroblast tissue, CEM cells, A549, and Vero cells [8]. Compared to epigallocatechin gallate (EGCG), major catechin extracted from green tea, TF is more stable under non-favorable conditions, making it a better candidate for antimicrobial agents [8]. TFDG was chosen based on a study indicating that TFDG was the most effective in inhibiting *Streptococcus mutans* (*S. mutans*) growth compared to TF, TF3G, and TF3′G [9]. Most previous studies focused on the antioxidant properties of theaflavin. It has been documented that drinking six cups of black tea could significantly increase the antioxidant capacity within the cell [6]. The number of polyphenols in tea varies highly depending on the origin and the brewing technique. Previous studies reported that the level of TFDG ranges from 0.07 to 1.13 g per 100 g of dry leaves among Darjeeling, Assam, Sri Lankan, African, and Chinese tea samples [10]. The level of TFs is approximately 7 mg when brewing a standard US tea bag (2.25 g) in 100 mL of water for less than 2 min and can increase to 14 mg when brewed for more than 4 min [11]. A study using LDL conjugation compared the antioxidant properties of theaflavin to green tea polyphenols, including epicatechin (EC), epicatechin gallate (ECG), epigallocatechin (EGC), and EGCG. The result showed that TFDG > ECG > EGCG ≥ TF3′G ≥ TF3G > TF ≥ EC > EGC in terms of antioxidant properties [12]. Based on the promising results, interest has expanded into the antiviral and antibacterial effects of TFs [8,13,14,15,16,17]. One study indicated 125 µg/mL TFs was the minimum inhibitory concentration (MIC) against *Porphyromonas gingivitis* (*P. gingivalis*) and 250 µg/mL TFDG in *Clostridium perfringens* (*C. perfringens*) while in Hepatitis C virus (HCV), 25 µg/mL TF3 was acting directly on the virus to prevent viral entry into the cell [13,18,19]. Several research groups also investigated the beneficial effect of TFs on the bacterial population and signaling pathways in the oral cavity and gut [20,21]. Some antibacterial mechanisms of TFs have been suggested, including reducing biosynthetic and metabolic activities in *C. perfringens* and anti-hemolytic activity in *Staphylococcus aureus* (*S. aureus*) [13,17].

This study explores the antimicrobial effect against nine pathogenic and clinically significant bacteria. Gram-negative *Klebsiella aerogenes* (*K. aerogenes*) is typically associated with nosocomial outbreaks due to the emergence of multidrug-resistant strains [22]. *Escherichia coli* (*E. coli*), a mutualism in the gastrointestinal (GI) tract of humans, is a model organism for Gram-negative bacteria [23,24,25,26,27,28,29]. *Pseudomonas aeruginosa* (*P. aeruginosa*), another nosocomial pathogen found among cystic fibrosis patients with a high mortality rate [30,31], has high resistance to most antimicrobial agents [30]. Multidrug-resistant *Proteus mirabilis* (*P. mirabilis*) is resistant to almost all antibiotic classes, and its prevalence among UTI infections has significantly increased [32].

The “*Bacillus cereus* group” includes several species of closely related pathogenic species like *Bacillus anthracis* (*B. anthracis*) and *Bacillus cereus* (*B. cereus*) [33]. *B. anthracis* and *B. cereus* are the causative agents of anthrax and the emetic syndrome, respectively [33]. *Bacillus subtilis* (*B. subtilis*) is considered a model organism for cellular development, including spore formation, germination, and biofilm production [34,35]. *Bacillus* spores may remain dormant for years but can germinate in favorable conditions such as specific nutrient reintroduction [36,37,38,39,40,41]. *Staphylococcus aureus* (*S. aureus*) causes a variety of life-threatening diseases, including endocarditis, toxic shock syndrome (TSS), and osteomyelitis [41]. Group A *Streptococcus pyogenes* (*S. pyogenes*) is a beta-hemolytic strain that can cause a wide range of infections, from superficial epithelial infection to the more severe streptococcal TSS (STSS) [42,43]. 

Acid-fast *Mycobacterium tuberculosis* (*M. tuberculosis*) is the pathogenic bacterium that causes tuberculosis (TB) [44]. Over the past three decades, TB has re-emerged as a global health concern, and in 2019, it is estimated that 10 million people were infected with TB worldwide, and 1.4 million people died [45]. Since detecting multidrug-resistant *M. tuberculosis* (MDR TB), research agencies, non-profit agencies, and academia have spared no effort to develop new treatments [46,47].

**Figure 1:**
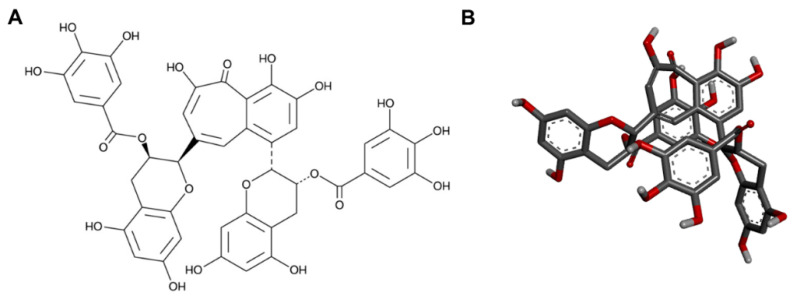
The (**A**) 2D and (**B**) 3D chemical structures of TFDG.

This study focuses on the antibacterial and sporicidal activity of TFDG as it contains the highest antioxidant [12] and antiviral [8,19] effects among all theaflavins. Each species used poses a public health threat. Most previous reports centered on the antibacterial activities of green tea polyphenols or a mixture of theaflavins against one or a few species. On the other hand, our results demonstrate that TFDG could potentially serve as a broad-spectrum antimicrobial agent that can inhibit the growth of nine bacteria across Gram-positive, Gram-negative, and acid-fast groups and an antispore agent. The anti-germination mechanism of TFDG against two *Bacillus* spp. is also proposed. Both 2D and 3D structures of TFDG are displayed in Figure 1. The 3D structure is used for molecular docking analysis.

## 2. Results

### 2.1. Determination of MIC and Half-Maximal Inhibitory Concentration (IC_50_)

No bacterial growth was observed when treated with 250 µg/mL or higher TFDG, so the MIC was determined as 250 µg/mL. The IC_50_ for all bacteria is around 62.5 µg/mL. As for erythromycin, the IC_50_ ranged from 7 to 26 µg/mL, and the MIC should be greater than 45 µg/mL for the bacteria tested in this study (Supplementary Figures S1–S3). 

### 2.2. Colony Forming Unit (CFU) Assay

Based on the microplate assay result, the effect of TFDG on the bacteria was further analyzed using CFU assay (Table 1). At the sixth hour, 62.5 µg/mL was able to inhibit the bacteria from 43.20% to 55.37% and ranged from 93.12% to 99.98% for 250 µg/mL TFDG. This correlates to the log reduction ranging from 0.25 to 0.35 for 62.5 µg/mL TFDG and from 1.17 to 3.69 for 250 µg/mL TFDG. Among nine bacteria tested, 250 µg/mL TFDG worked the best on *P. aeruginosa* (99.98 ± 0.01%). All the data were statistically significant (*p* < 0.05).

### 2.3. BacTiter-Glo^TM^ Microbial Cell Viability Assay

62.5 µg/mL TFDG was able to inhibit up to 59.82 ± 6.19% of cell viability based on the ATP level compared to the control, while 250 µg/mL TFDG was able to inhibit up to 99.33 ± 0.16% of bacteria (Table 2).

### 2.4. Live/Dead Bacterial Viability Assay

**Figure 2:**
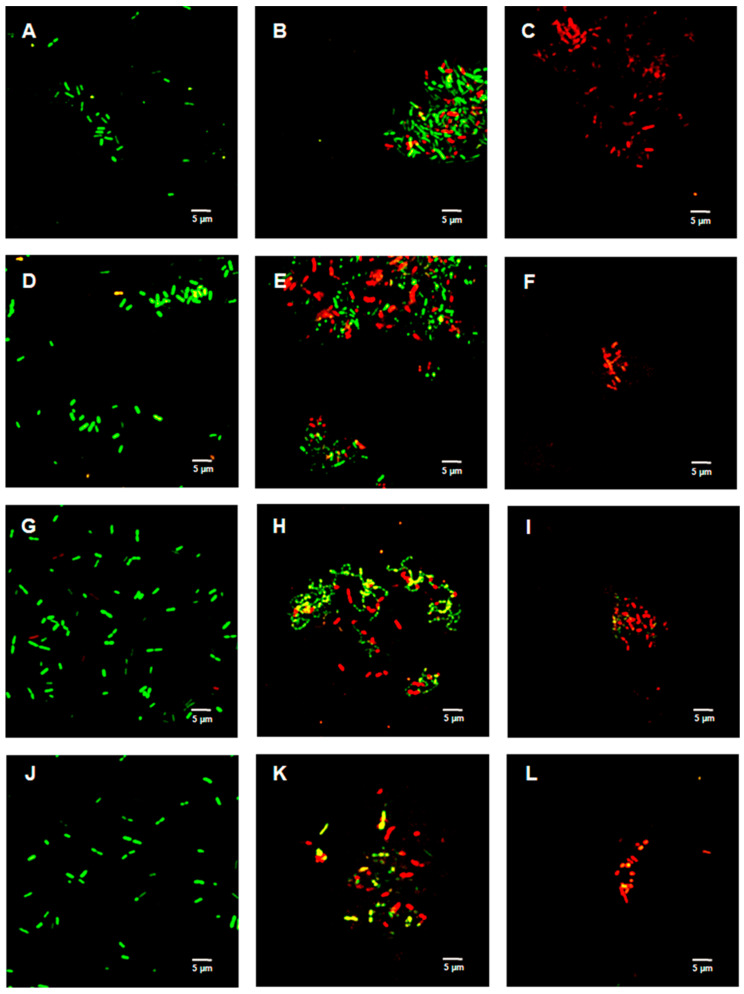
Live/Dead assay of Gram-negative species with various concentrations of TFDG at 6-h incubation. The samples were visualized using Olympus confocal microscope. The green indicates live bacteria, while the red indicates dead bacteria. (**A**) *K. aerogenes* control; (**B**) *K. aerogenes* with 62.5 µg/mL TFDG; (**C**) *K. aerogenes* with 250 µg/mL TFDG; (**D**) *E. coli* control; (**E**) *E. coli* with 62.5 µg/mL TFDG; (**F**) *E. coli* with 250 µg/mL TFDG; (**G**) *P. aeruginosa* control; (**H**) *P. aeruginosa* with 62.5 µg/mL TFDG; (**I**) *P. aeruginosa* with 250 µg/mL TFDG; (**J**) *P. mirabilis* control; (**K**) *P. mirabilis* with 62.5 µg/mL TFDG; and (**L**) *P. mirabilis* with 250 µg/mL TFDG.

**Figure 3:**
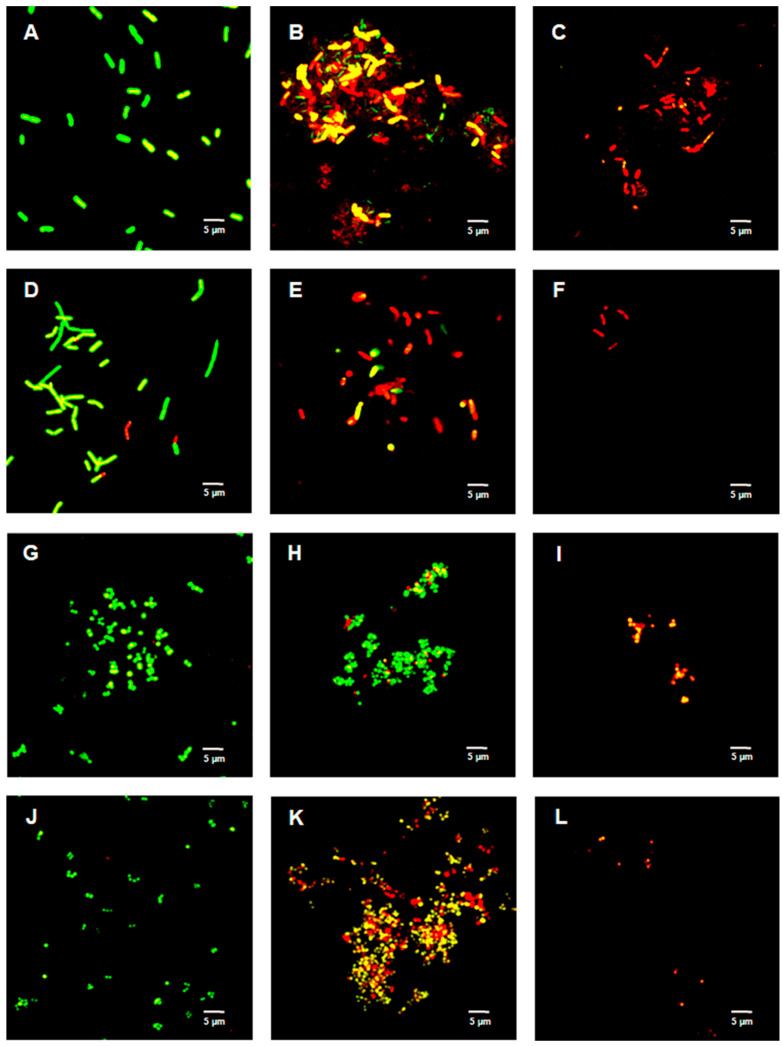
Live/Dead assay of Gram-positive species with various concentrations of TFDG at 6-h incubation. The samples were visualized using Olympus confocal microscope. The green indicates live bacteria, while the red indicates dead bacteria. (**A**) *B. cereus* control; (**B**) *B. cereus* with 62.5 µg/mL TFDG; (**C**) *B. cereus* with 250 µg/mL TFDG; (**D**) *B. subtilis* control; (**E**) *B. subtilis* with 62.5 µg/mL TFDG; (**F**) *B. subtilis* with 250 µg/mL TFDG; (**G**) *S. aureus* control; (**H**) *S. aureus* with 62.5 µg/mL TFDG; (**I**) *S. aureus* with 250 µg/mL TFDG; (**J**) *S. pyogenes* control; (**K**) *S. pyogenes* with 62.5 µg/mL TFDG; and (**L**) *S. pyogenes* with 250 µg/mL TFDG.

**Figure 4:**
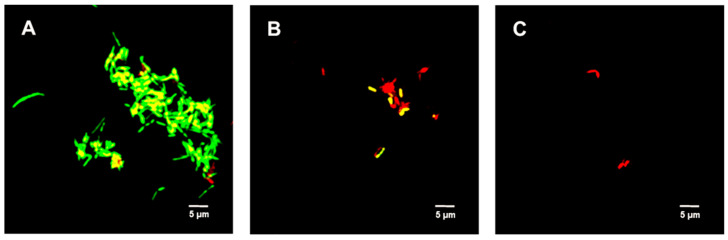
Live/Dead assay of *M. smegmatis* with various concentrations of TFDG at 6-h incubation. The samples were visualized using Olympus confocal microscope. The green indicates live bacteria, while the red indicates dead bacteria. (**A**) *M. smegmatis* control; (**B**) *M. smegmatis* with 62.5 µg/mL TFDG; and (**C**) *M. smegmatis* with 250 µg/mL TFDG.

Figure 2, Figure 3 and Figure 4 show the bacterial viability when treated with various concentrations of TFDG after 6 h. Overall, the control bacteria were primarily green and maintained normal morphology. Cells treated with 62.5 µg/mL TFDG showed a mixture of live, impaired, and dead cells. Most cells appeared to be smaller and more clumped together when compared to the control except for the *M. smegmatis*, which had less aggregation. Cells treated with 250 µg/mL TFDG were mainly non-viable. The cell numbers were significantly less than the control. The cell morphology appeared smaller and/or segmented with TFDG treatment.

### 2.5. Germination Inhibition via CFU Assay 

The percent (%) inhibition was calculated from the CFU assay after a 60-min TFDG treatment. 312.5 µg/mL TFDG inhibited the *Bacillus* spores from germinating, ranging from 54.13% to 60.49%, while 625 µg/mL TFDG was able to inhibit germination ranging from 99.37% to 99.92% (Table 3). 

### 2.6. Live/Dead Spore Viability Assay

**Figure 5:**
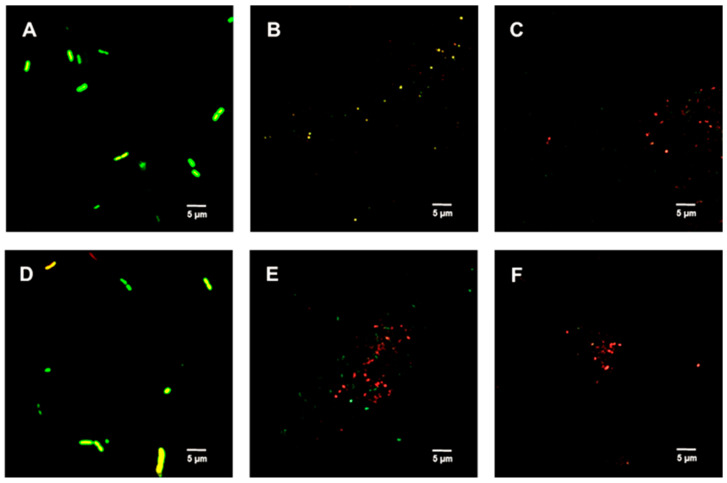
Live/Dead assay of *Bacillus* spp. spores with TFDG at 60-min incubation. The green indicates viable spores, while the red indicates non-viable spores. (**A**) *B. cereus* spores control; (**B**) *B. cereus* spores with 312.5 µg/mL TFDG; (**C***) B. cereus* spores with 625 µg/mL TFDG; (**D**) *B. subtilis* spores control; (**E**) *B. subtilis* spores with 312.5 µg/mL TFDG; and (**F**) *B. subtilis* spores with 625 µg/mL TFDG.

Figure 5 shows the untreated (control) spores were primarily green. Samples treated with 312.5 µg/mL TFDG indicated the spores were impaired, while 625 µg/mL TFDG-treated spores were mainly non-viable.

### 2.7. Binding Pocket

The protein structures related to the four genes of interest were analyzed via CASTp to determine its binding pocket. Table 4 shows the binding pocket areas (Å**^2^**) for GPR were 616.45 and 433.40 for *B. cereus* and *B. subtilis*, respectively. The binding pocket areas (Å**^2^**) for Lgt were 1284.21 for *B. cereus* and 1565.20 for *B. subtilis*. The amino acid residues of each pocket were used as a guide to determine the location and size of the grid for *in silico* docking analysis. 

### 2.8. In Silico Docking Analysis

**Figure 6:**
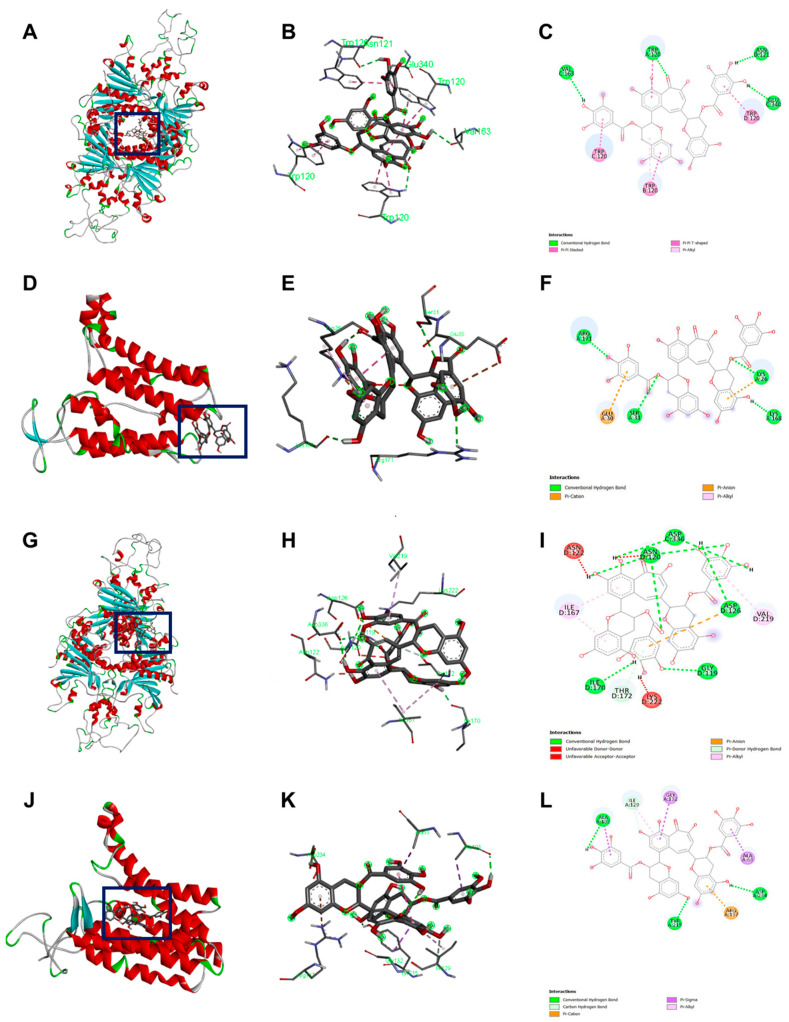
Docking visualization of TFDG with the germination protein using BIOVIA Discovery Studio Visualizer. (**A**–**C**) *B. cereus* GPR with TFDG; (**D**–**F**) *B. cereus* Lgt with TFDG; (**G**–**I**) *B. subtilis* GPR with TFDG; (**J**–**L**) *B. subtilis* Lgt with TFDG. For (**C**,**F**,**I**,**L**) the bright green line shows conventional hydrogen bond; bright pink shows the pi-pi interaction; light pink shows pi-alkyl interaction; orange shows pi cation, pi carbon, and pi-anion interaction; the red line shows unfavorable bond interaction; light green shows pi-donor hydrogen interaction and carbon-hydrogen interaction; and bright purple shows pi sigma interaction.

Table 5 details the molecular docking result of TFDG for both GPR and Lgt in *B. cereus* and *B. subtilis*. The *B. cereus* GPR docking score was −9.7 kcal/mol with five bonds, including conventional H-bond and Pi-Stacked. *B. cereus* Lgt binding score with TFDG was −7.6 kcal/mol with five different bonds that include conventional H-bond and pi-cation. Both GPR and Lgt of *B. subtilis* had the same binding score of −10.3 kcal/mol, but GPR has 12 bonds, including conventional H-bond, unfavorable donor-donor, and pi-donor H-bond. In contrast, Lgt has only seven bonds, including hydrophobic pi-sigma, conventional H bond, and carbon H bond. The binding pocket and the bond for each protein were observed using Discovery Studio, as seen in Figure 6. 

Previous studies showed the −7.0 kcal/mol threshold as significant for AutoDock binding [48,49]. Since both proteins showed a higher negative value, it indicates these two are good candidates for TFDG anti-germination properties evaluation. Hydrogen bond regulates molecular interaction through donor-acceptor pairing, enhancing receptor-ligand interaction [50]. Instead, hydrophobic interaction is a major consideration for binding affinity as this interaction can be considered a weak hydrogen bond [50]. *B. cereus* GPR binding affinity consists of 4 hydrogen bond interactions, while Lgt (GerF) consists of four hydrogen interactions. *B. subtilis* GPR TFDG binding affinity consists of nine hydrogen bonds interactions, while Lgt (GerF) has four hydrogen bonds and three strengthening hydrophobic interactions. 

### 2.9. Semi-Quantitative RT-PCR

**Figure 7:**
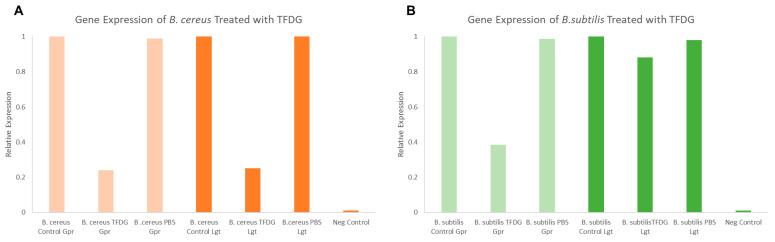
Semi-quantitative RT-PCR *lgt* and gpr gene expression analysis of *Bacillus* spore when treated with TFDG. (**A**) *B. cereus* spores; (**B**) *B. subtilis* spores.

Figure 7 shows the relative expression of both *lgt* and *gpr* after a one-hour treatment of 625 µg/mL TFDG. In both *B. cereus* and *B. subtilis*, the expression of *gpr* dropped to 0.20 and 0.39, respectively, compared to the control (1.00). On the other hand, the expression of *lgt* was lower only in *B. cereus* (0.25) but not in *B. subtilis* (0.88) when compared to the control (1.00).

## 3. Discussion

The urgency to find a novel antimicrobial agent has pushed researchers to look for either natural or synthetic alternatives. Currently, there are 42 new antibiotics under clinical development, but only 11 can treat pathogens that are considered critical by the World Health Organization (WHO) [51]. Antibiotic development projects from major pharmaceutical companies only account for four out of 42 studies, focusing on more profitable ventures like immune-oncology therapeutics [51]. For this reason, it is time for us to seek alternative solutions to ease the healthcare and economic burden of developing new antibiotics. 

In this study, several methods were used to demonstrate the effectiveness of TFDG in inhibiting cell growth. As seen in Figure 2, Figure 3 and Figure 4 and Table 1 and Table 2, 250 µg/mL TFDG consistently inhibits ≥ 90% of cells compared to the control at 6-h incubation. BacTiter-Glo^TM^ measures the ATP level in the cell since extracellular ATP peaked at the end of the log phase but decreased during the stationary phase [52]. This test is more sensitive as it directly detects the presence of the ATP level in the sample, indicating the metabolically active viable cells, which do not discriminate between live and dead cells [53]. The Live/Dead assay measures the permeability of the cellular membrane. Red-colored cells indicate the cell membranes were damaged when treated with TFDG (Figure 2, Figure 3 and Figure 4). These two assays provided further proof that TFDG could effectively inhibit bacterial growth. Ignasimuthu et al. [54] showed the MIC of EGCG for *B. subtilis*, *E. coli*, and *S. aureus* ranging from 130 to 580 μg/mL via broth dilution method. This suggests that TFDG might be comparable or even better as an antibacterial agent when compared to EGCG. Further studies of the effects of TFDG against clinically significant strains like ESKAPE (*Enterococcus faecium*, *S. aureus*, *K. pneumoniae*, *A. baumannii*, *P. aeruginosa*, and *Enterobacter* spp.) bacteria and other drug-resistant strains such as MRSA, VRSA, carbapenem-resistant Enterobacterales (CRE) will be carried out [55]. In terms of the antibacterial mechanism, TFDG decreased the eDNA and dextran production in *S. mutans* while decreasing the expression nucleoid synthesis in *Clostridium perfringens* (*C. perfringens*) [9,13]. A recent transcriptome study also shows that TFs (80% purity) could inhibit different virulence factors, including glucosyltransferases, gtfB, gtfC, and gtfD in *S. mutans*. The antimicrobial mechanism of TFDG on other species remains undetermined [56].

Bacterial spores are associated with foodborne diseases and food spoilage, and human diseases like gas gangrene, anthrax, and botulism [36]. Table 3 and Figure 5 show the successful inhibition of both *B. cereus* and *B. subtilis* from germinating. This is a promising observation, as TFDG could still prevent the spore from germinating above 99% for both species. The ability of EGCG to inhibit sporulation is well-documented across *Bacillus* spp. [16,57]. The findings in this study provide further evidence that tea polyphenols could serve as a potent antimicrobial agent. CASTp is an online tool that analytically predicts pocket cavities by utilizing the algorithmic and theoretical modeling that excludes shallow depression [58]. This binding pocket was utilized to determine the binding affinity of TFDG. Molecular docking in this study helps understand the mechanism of TFDG. Previous findings showed that compounds with binding energies of −7.0 kcal/mol or less are considered significant [48]. This threshold eliminates either weak or non-specific binding energies [49]. Chang et al. [49] showed that this threshold could detect 98% of known inhibitors of HIV therapeutics. This threshold has also been shown to eliminate 95% of non-inhibitor interaction [49]. This study uses AutoDock Vina for binding analysis. It is a freely accessible tool that best performed in predicting high-affinity ligands and showed the most consistent performance in a study by Kukol [59]. Table 4 and Figure 6 show that the binding affinity of TFDG for *B. cereus* GPR and Lgt were −9.7 and −7.6 kcal/mol, respectively. As for *B. subtilis*, TFDG affinity for GPR and Lgt were the same, at −10.3 kcal/mol. Based on the promising binding results, a semi-quantitative RT-PCR was carried out to investigate the relative expression of both genes when treated with TFDG. This method is sensitive and reliable in detecting limited transcripts from the samples [60]. The result in Figure 7 shows that the relative expression of *gpr* was significantly lower (0.20 to 0.39) than the control in both *B. cereus* and *B. subtilis*. The relative expression of *lgt* was higher in *B. subtilis* (0.88) than *B. cereus* (0.25) compared to the control. Overall, TFDG may affect both *lgt* and *gpr* expression in *B. cereus* while only *gpr* expression in *B. subtilis*. The GPR protease encoded by *gpr* is a germination protease in the spore coat, responsible for degrading the small acid-soluble protein (SASPs) [61]. This is a conserved gene in *Bacillus* spp. *Clostridium* spp. and *Clostridiodides* spp. involving in protein synthesis and energy metabolism for early spore outgrowth [61,62]. A conserved gene, *lgt* or *gerF* in *Bacillus* spp. and *Clostridium* spp. codes for prelipoprotein diacylglycerol transferase [62,63]. This enzyme acts as a catalyst for the transfer of diacylglycerol to a cysteine residue in bacterial membrane prelipoproteins [63]. Mutation in this gene results in a slower germination process even in a favorable environment [63,64]. The deletion of *lgt* in *B. anthracis* causes a decrease in surface hydrophobicity that eventually leads to lower virulence in the mutant strain [64]. In *B. subtilis*, the mixture of Ca^2+^ and dipicolinic acid (Ca-DPA) complex with GerF occurs during germination [63]. Li et al. [65] reported that the binding dissociation constant (Kd) between Ca-DPA and its native ligand SpoVAD was 0.8. To the best of our knowledge, this is the first study that investigates the binding affinity between Ca-DPA and GerF in *Bacillus* spp. Both GPR and GerF show favorable outcomes in inhibiting the germination process from *in silico* analysis. The favorable binding affinity, along with multiple numbers of hydrogen and hydrophobic bonds, suggests that these two proteins could be the potential targets of TFDG in inhibiting the germination process. Semi-quantitative results support that TFDG inhibits the expression of the conserved genes. Thus, TFDG should be further investigated as a natural food additive. 

Figure 2, Figure 3 and Figure 4 show the cells clumped together, the self-binding process known as auto-agglutination/auto-aggregation [66]. This is a widely observed phenomenon and is considered the first step in biofilm formation [66]. Auto-aggregation occurs under stressful conditions, such as temperature change, and protects the cells from external stressors [66]. Generally, auto-aggregation is mediated by surface proteins like the self-associating autotransporters (SAATs) in *Enterobacteriaceae* [66]. In *Actinobacillus pleuropneumoniae* (*A. pleuropneumoniae*), the adhesin gene *adh* was involved in the biofilm formation, and the deletion of this gene could decrease pathogenicity [67]. Similarly, the first steps of biofilm formation in *Helicobacter pylori*, *P. gingivalis*, and *Staphylococcus epidermidis* are also through auto-aggregation via their adhesin genes [68,69,70]. The effects of TFDG, especially pertaining to adhesion and biofilm formation, would be a pivotal step to better understanding the antimicrobial mechanism of TFDG. 

## 4. Materials and Methods

### 4.1. Bacteria Culture

The bacterial cultures used in this study include Gram-negative: *Klebsiella aerogenes* (*K. aerogenes*) (155030A), *Escherichia coli* (*E. coli*) (155065A), *Pseudomonas aeruginosa* (*P. aeruginosa*) (155250A), and *Proteus mirabilis* (*P. mirabilis*) (155239A); Gram-positive: *Bacillus cereus* (*B. cereus*) (154870A), *Bacillus subtilis* (*B. subtilis*) (154921A), *Staphylococcus aureus* (*S. aureus*) (155554A), and *Streptococcus pyogenes* (*S. pyogenes*) (155630A); and acid-fast *Mycobacterium smegmatis* (*M. smegmatis*) (155180A). All cultures were obtained from Carolina Biological (Carolina Biological, Burlington, NC, USA).

### 4.2. Culture Maintenance

All cultures were maintained in tryptic soy broth (TSB) or tryptic soy agar (TSA) except for *S. pyogenes* and *M. smegmatis*, which were maintained in brain heart infusion broth (BHIB) (Bacto^TM^, Sparks, MD, USA). The media were made with Milli-Q Integral 5 Water Purification System (Millipore Sigma, Billerica, MA, USA) based on the manufacturer’s protocol. All experiments were performed using fresh overnight culture. The purity of the cultures was routinely checked.

### 4.3. Theaflavin Preparation

Theaflavins were obtained from a nutraceutical company (DH Nutraceuticals, LLC, Edison, NJ, USA). Theaflavin-3,3′-digallate (TFDG) was extracted and purified using ethyl acetate fraction, LH-20 column, 40% acetone solution elution then concentrated via rotary evaporator [71]. Purified 10 mg/mL TFDG stock solution was prepared with 200 proof ethanol (EtOH) (DLI, King of Prussia, PA, USA). The theaflavin stock was diluted in bacterial growth media to the desired concentrations accordingly. 

### 4.4. Microplate Assay

The bacterial growth was monitored with different TFDG concentrations (0, 62.5, 125, and 250 µg/mL) over a 12-h period. In a 96-well plate, 10 µL of overnight culture (OD_600nm_ = 1.0) was added to each well along with various concentrations of TFDG and TSB to yield a final volume of 120 µL. The optical density was recorded hourly using a Varioskan™ LUX multimode microplate reader and analyzed via SkanIt Software (Thermo Scientific^TM^, Waltham, MA, USA). The positive control was 10% bleach, while bacterial growth media was used as the negative control. The highest solvent concentration (1% EtOH) was also tested. Erythromycin, a broad-spectrum antibiotic, has also been included as a reference molecule for antibacterial efficacy comparison. The experiments were performed in triplicate. The microplate assay results established the half-maximal inhibitory concentration (IC_50_) and minimum inhibitory concentration (MIC). The lowest concentration with no bacterial growth was defined as MIC. The IC_50_ was calculated based on a dose-response curve with log (concentration) as the x-axis and percent inhibition as the y-axis based on 0, 62.5, 125, and 250 µg/mL. The concentration that correlates to the 50% inhibition is the IC_50_.

### 4.5. Colony Forming Unit (CFU) Assay

Following the microplate assay, the cultures treated with 0, 62.5, and 250 µg/mL TFDG were collected after 6-h incubation, serially diluted (from 10^−2^ to 10^−8^) plated on TSA. The plates were incubated for 12 h at 37 °C, and the experiments were done in triplicate. The CFUs were recorded, and the percent inhibition was calculated based on the following formula:

(1) $$Percent\;Inhibition=\left[\frac{CFU_{untreated}\;-CFU_{treated}}{CFU_{untreated}}\right]\times 100$$

The log reduction of the CFU was also calculated based on the following formula:

(2) $$Log\;Reduction=Log10\;\left(\frac{CFU_{untreated}}{CFU_{treated}}\right)$$

### 4.6. BacTiter-Glo^TM^ Microbial Cell Viability Assay

BacTiter-Glo^TM^, the luciferase-based assay that quantifies the amount of ATP of metabolically active cells, was conducted according to the manufacturer’s protocol (Promega, Madison, WI, USA) [54]. The reagent was prepared by mixing the BacTiter-Glo^TM^ buffer with the BacTiter-Glo^TM^ lyophilized substrate at room temperature. The mixture was then homogenized and incubated at room temperature for 15 min. 

In a black 96-well plate, the bacteria were prepared based on the microplate assay (TFDG concentration of 0, 62.5, and 250 µg/mL) and placed in an IS-500 Incubator Shaker (Chemglass Life Sciences LLC, Vineland, NJ, USA) at 37 °C, 250 rpm for six hours. Then 120 µL of the BacTiter-Glo^TM^ reagent was added to each well. The plate was wrapped in aluminum foil and placed in the incubator shaker for five minutes. The luminescence was read using a Varioskan™ LUX multimode microplate reader and analyzed via SkanIt Software (Thermo Scientific^TM^, Waltham, MA, USA). The experiments were done in triplicate. The percent inhibition was calculated based on the following formula:

(3) $$Percent\;Inhibition=\left[\frac{\left(RFU_{untreated}\;-RFU_{treated}\right)}{RFU_{untreated}}\;\right]\times 100$$

The log reduction of the RFU was also calculated based on the following formula:

(4) $$Log\;Reduction=Log10\;\left(\frac{RFU_{untreated}}{RFU_{treated}}\right)$$

### 4.7. LIVE/DEAD^TM^ BacLight^TM^ Bacterial Viability Assay

The Live/Dead Viability is a two-dye system consisting of Syto9 green fluorescent dye and propidium iodide (PI) red fluorescent dye. Both nucleic acid dyes can be used to differentiate live from dead bacteria. PI penetrates damaged bacterial membranes while Syto9 stains bacteria with intact cell membranes. Thus, live cells will be stained in green, impaired cells in yellow, and dead cells in red. 

The staining was done using the Invitrogen^TM^ Live/Dead BacLight^TM^ Bacterial Viability Kit. According to the manufacturer’s recommendation, equal parts of the Syto9 and PI were combined (Thermo Fisher Scientific, Waltham, MA, USA). 

Following the same experimental setup as the CFU assay mentioned above (0, 62.5, and 250 µg/mL TFDG), the dye mixture was added to each culture and incubated at room temperature in the dark for 15 min. The cells were then observed using Olympus IX81 FV1000 Confocal Microscope, and the images were analyzed using the FV10-ASW 4.2 viewer. This method was also utilized to visualize the germinated spores, except the spores were treated for 60 min.

### 4.8. Spore Preparation

This method was modified based on the previously published protocol [16,72]. *B. cereus* and *B. subtilis* are spore-forming bacteria. The spores were induced by adding 5 mL of fresh overnight culture (OD_600nm_ = 1.0) to 5 mL sterile diH_2_O in a culture tube. The cultures were incubated at 37 °C and 250 rpm for 72 h (IS-500 Incubator Shaker, Chemglass Life Sciences LLC, Vineland, NJ, USA). After 72 h, the spores were heated for 20 min at 75 °C to inactivate the vegetative cells. The purity of the spores was confirmed through the Schaeffer Fulton differential stain method.

### 4.9. Spore Germination Inhibition Assay

100 µL of the 72-h spores were added to various concentrations of TFDG (312.5 µg/mL and 625 µg/mL) along with TSB to a final volume of 1 mL in a microcentrifuge tube. The tubes were incubated for 1 h at 37 °C and 250 rpm (IS-500 Incubator Shaker, Chemglass Life Sciences LLC, Vineland, NJ, USA). After the incubation period, the samples were serially diluted (from 10^−2^ to 10^−9^), and 100 µL of the dilution was plated using the spread plate method with TSA. The plates were incubated for 12 h at 37 °C. The experiments were done in triplicate. The CFUs were recorded, and the percentage of inhibition and log reduction was calculated based on the formula above (1 and 2).

### 4.10. Ligand Preparation

The 2D structure of TFDG (structure ID: 135403795) was obtained from PubChem (https://pubchem.ncbi.nlm.nih.gov/, accessed on 27 August 2021). The structure was converted into 3D format using Vega ZZ software (http://www.vegazz.net, accessed on 27 August 2021) and .pdbqt file format using AutoDock Tools V1.5.6 [73]. 

### 4.11. Gene and Protein Selection

The germination genes for *in silico* modeling were selected based on conserveness in *Bacillus* spp. and *Clostridium* spp. [74]. The genes and their protein information were tabulated in Table 6. The protein structure of each gene was downloaded directly from PDB (https://www.rcsb.org/, accessed on 3 September 2021) for the binding analysis. If the protein structure was not yet crystalized, the protein sequence was used to construct a hypothetical structure using SwissModel (https://swissmodel.expasy.org/, accessed on 3 September 2021) [75]. The hypothetical structure with the highest sequence identity was chosen for the analysis. The binding pocket was then determined using the Computed Atlas of Surface Topography of Proteins (CASTp) (http://sts.bioe.uic.edu/castp/, accessed on 3 September 2021) [58].

### 4.12. In Silico Docking Analysis and Visualization

*In silico* docking analysis was performed using AutoDock Vina. The size and search space of each protein were calculated using AutoDock Tools 1.5.6 based on the result from the CASTp analysis. Table 7 shows the grid box for each analysis. The spacing was left at the default value of 0.375 Å, as well as the exhaustiveness rate of 8 [73].

### 4.13. Total RNA Extraction, cDNA Synthesis, and Semi-Quantitative RT-PCR

The spore germination assay was carried out for control, PBS, and 625 µg/mL TFDG. After one hour of treatment, the RNA extraction was done using the Ambion^®^ RiboPure^TM^ Kit (Ambion Inc, Austin, TX, USA). Then 250 μL of the sample was mixed with the 1 mL TRI reagent. The mixture was sonicated 15 times at 3 s intervals (20% power) using the Branson Sonifier Cell Disruptor 200 (Emerson Industrial, St. Louis, MO, USA). The extraction process was carried out based on the manufacturer’s protocol. The RNA was used as a template for the cDNA synthesis using the ABI High Capacity cDNA Reverse Transcription Kit (Applied Biosystems-Life Technologies, Camarillo, CA, USA). The cDNA synthesis was done according to the manufacturer’s protocol. The cDNA synthesis was carried out in a Veriti 96-Well Thermocycler (Applied Biosystems, Camarillo, CA, USA). The cDNA purity and concentration were measured using a BioDrop uLite (Biochrom, Cambridge, United Kingdom). The samples were stored at −20 °C. 

The oligonucleotides were designed based using NCBI Primer Design Tool (https://www.ncbi.nlm.nih.gov/tools/primer-blast/, accessed 3 November 2021). The following primers were generated: Bc_GPR_F2: 5′-ACACCAGATGCTCTTGGACC-3′ and Bc_GPR_R2: 5′-TCTGCTCTTTTCATCCGGCA-3′; Bc_Lgt_F2: 5′-CTGTATGGGCTTTTGGGGCA and Bc_Lgt_R2: 5′-TGAGCAAACCCCTCAACAAT-3′; Bs_GPR_F2: 5′-CCGATTTGGCAGTGGAAACG-3′ and Bs_GPR_R2: 5′-AACACCAAGCGTGTCTTTGC-3′; Bs_Lgt_F2: 5′-TTTGCTCGGGCTGTGGATAG-3′ and Bs_Lgt_R2 5′ CCCTTGACGCGTCTGAAGAT-3′; 16S ribosomal RNA 27F: 5′-AGAGTTTGATCCTGGCTCAG-3′ and 1492R: 5′-ACGGCTACCTTGTTACGACTT-3′. The ~100 µg/mL cDNA was used, and the PCR was performed using the Applied Biosystems Veriti 96-Well Thermal Cycler (ThermoFisher Scientific, MA, USA): 95 °C for 60 s, 35 cycles of 95 °C for 10 s, 56 °C for 10 s and 72 °C for 90 s. A 2% agarose gel electrophoresis was carried out, and the amount of cDNA was determined [76]. The relative mRNA expression was then calculated.

### 4.14. Statistical Analysis

All experiments were performed in triplicate, and the mean and standard deviations (SD) were calculated. One-way Analysis of Variance (ANOVA) and Dunnett’s post hoc analysis were used to analyze the data (GraphPad Prism 5, San Diego, CA, USA). A *p*-value less than 0.05 was considered statistically significant.

## 5. Conclusions

This study profiled the effects of TFDG on nine bacteria, including Gram-positive, Gram-negative, and acid-fast bacteria. Microplate assay and the CFU assay were carried out. The microplate assay results indicated the MIC was 250 µg/mL. BacTiter-Glo^TM^ Microbial Cell Viability test was also performed to measure the level of ATP in the sample. The fluorescence-based Live/Dead Assay was utilized to visualize the morphological changes on individual cells to TFDG, thus precluding the possible antibacterial mechanism of TFDG. *In silico* modeling allowed us to analyze and propose the mechanism of TFDG on the bacterial spores at the molecular level. Semi-quantitative RT-PCR assays were carried out for gene expression analysis pre- and post-treatment. This study successfully shows the potential usage of TFDG as an antimicrobial agent for a wide selection of bacteria, ranging through Gram-negative, Gram-positive, and acid-fast species. This study also shows the anti-germination properties of TFDG as TFDG inhibits the expression of *gpr* and *lgt*, genes code for the conserved GPR and Lgt (GerF) germination proteins based on *in silico* modeling and semi-quantitative RT-PCR.

## Figures and Tables

**Table 1:** Colony-forming unit (CFU/mL) with the log reduction and percent inhibition of different bacteria with TFDG.

		Bacteria	TFDG (µg/mL)	CFU/mL (Mean ± SD)	Log Reduction (Mean ± SD)	% Inhibition (Mean ± SD)
		*K. aerogenes*	0	(9.86 ± 0.09) × 10^8^	0	0
Gram-negative			62.5	(4.40 ± 0.12) × 10^8^	0.35 ± 0.02	55.37 ± 1.59%
			250	(6.07 ± 0.48) × 10^7^	1.17 ± 0.03	93.12 ± 0.46%
		*E. coli*	0	(1.09 ± 0.06) × 10^9^	0	0
			62.5	(6.20 ± 0.42) × 10^8^	0.25 ± 0.01	43.20 ± 0.83%
			250	(2.36 ± 0.66) × 10^7^	1.69 ± 0.12	97.87 ± 0.52%
		*P. aeruginosa*	0	(7.01 ± 0.07) × 10^8^	0	0
			62.5	(3.68 ± 0.11) × 10^8^	0.28 ± 0.01	47.50 ± 1.15%
			250	(1.57 ± 0.60) × 10^5^	3.69 ± 0.21	99.98 ± 0.01%
		*P. mirabilis*	0	(6.27 ± 0.08) × 10^9^	0	0
			62.5	(3.17 ± 0.05) × 10^9^	0.30 ± 0.00	49.39 ± 0.23%
			250	(1.57 ± 0.39) × 10^7^	2.62 ± 0.13	99.75 ± 0.07%
Gram-positive	Spore former	*B. cereus*	0	(6.54 ± 0.13) × 10^8^	0	0
			62.5	(3.32 ± 0.08) × 10^8^	0.29 ± 0.01	49. 16 ± 1.65%
			250	(6.00 ± 1.63) × 10^5^	3.05 ± 0.13	99.91 ± 0.03%
		*B. subtilis*	0	(6.89 ± 0.09) × 10^8^	0	0
			62.5	(3.35 ± 0.08) × 10^8^	0.31 ± 0.01	51.40 ± 0.72%
			250	(3.33 ± 0.82) × 10^5^	3.34 ± 0.13	99.95 ± 0.01%
	Non-spore former	*S. aureus*	0	(4.69 ± 0.12) × 10^9^	0	0
			62.5	(2.10 ± 0.18) × 10^9^	0.35 ± 0.04	55.20 ± 4.62%
			250	(3.03 ± 1.23) × 10^6^	3.24 ± 0.24	99.93 ± 0.03%
		*S. pyogenes*	0	(4.33 ± 0.02) × 10^9^	0	0
			62.5	(2.24 ± 0.05) × 10^9^	0.29 ± 0.01	48.28 ± 0.85%
			250	(3.47 ± 0.09) × 10^6^	3.10 ± 0.01	99.92 ± 0.05%
Acid-fast		*M. smegmatis*	0	(4.11 ± 0.07) × 10^9^	0	0
			62.5	(2.02 ± 0.05) × 10^9^	0.31 ± 0.02	50.88 ± 1.92%
			250	(1.33 ± 0.34) × 10^6^	3.50 ± 0.11	99.97 ± 0.01%

**Table 2:** Relative fluorescence unit (RFU) with the log reduction and percent inhibition of different bacteria treated with TFDG based on BacTiter-Glo^TM^ assay.

		Bacteria	TFDG (µg/mL)	RFU (Mean ± SD)	Log Reduction (Mean ± SD)	% Inhibition (Mean ± SD)
		*K. aerogenes*	0	(1.90 ± 0.46) × 10^6^	0	0
Gram-negative			62.5	(9.17 ± 2.01) × 10^5^	0.31 ± 0.01	51.52 ± 1.63%
			250	(1.51 ± 0.73) × 10^4^	2.14 ± 0.12	99.25 ± 0.22%
		*E. coli*	0	(1.67 ± 0.09) × 10^6^	0	0
			62.5	(7.89 ± 1.08) × 10^5^	0.33 ± 0.04	53.00 ± 4.48%
			250	(1.15 ± 0.19) × 10^4^	2.17 ± 0.09	99.31 ± 0.14%
		*P. aeruginosa*	0	(1.67 ± 0.36) × 10^6^	0	0
			62.5	(8.07 ± 1.91) × 10^5^	0.32 ± 0.02	51.97 ± 2.74%
			250	(6.16 ± 2.84) × 10^4^	1.48 ± 0.30	95.87 ± 2.39%
		*P. mirabilis*	0	(1.52 ± 0.05) × 10^6^	0	0
			62.5	(7.05 ± 0.36) × 10^5^	0.34 ± 0.02	53.77 ± 1.69%
			250	(3.52 ± 2.45) × 10^4^	1.78 ± 0.37	97.71 ± 1.58%
Gram-positive	Spore former	*B. cereus*	0	(2.03 ± 1.00) × 10^6^	0	0
			62.5	(7.58 ± 2.80) × 10^5^	0.40 ± 0.07	59. 82 ± 6.19%
			250	(1.28 ± 0.48) × 10^5^	1.18 ± 0.08	93.24 ± 1.26%
		*B. subtilis*	0	(2.30 ± 0.32) × 10^6^	0	0
			62.5	(1.09 ± 0.21) × 10^5^	0.33 ± 0.03	52.81 ± 3.47%
			250	(4.82 ± 1.41) × 10^4^	1.69 ± 0.07	97.94 ± 0.35%
	Non-spore former	*S. aureus*	0	(7.74 ± 2.02) × 10^6^	0	0
			62.5	(3.66 ± 1.08) × 10^6^	0.33 ± 0.03	53.12 ± 3.11%
			250	(8.55 ± 3.73) × 10^4^	1.98 ± 0.29	98.71 ± 0.76%
		*S. pyogenes*	0	(4.43 ± 0.99) × 10^6^	0	0
			62.5	(2.15 ± 0.42) × 10^6^	0.31 ± 0.05	50.81 ± 5.81%
			250	(1.01 ± 0.80) × 10^5^	1.83 ± 0.50	97.72 ± 1.41%
Acid-fast		*M. smegmatis*	0	(1.83 ± 0.25) × 10^6^	0	0
			62.5	(9.09 ± 0.59) × 10^5^	0.30 ± 0.04	49.82 ± 4.42%
			250	(1.24 ± 0.42) × 10^4^	2.19 ± 0.12	99.33 ± 0.16%

**Table 3:** Germination inhibition assay of both *B. cereus* and *B. subtilis*.

		Bacteria	TFDG (µg/mL)	CFU/mL (Mean ± SD)	Log Reduction (Mean ± SD)	% Inhibition (Mean ± SD)
Gram-positive	Spore former	*B. cereus*	0	(1.11 ± 0.85) × 10^10^	0	0
			312.5	(4.79 ± 3.55) × 10^9^	0.34 ± 0.03	54. 13 ± 3.51%
			625	(6.55 ± 4.57) × 10^5^	2.22 ± 0.12	99.37 ± 0.17%
		*B. subtilis*	0	(7.24 ± 4.90) × 10^9^	0	0
			312.5	(3.25 ± 2.33) × 10^9^	0.41 ± 0.09	60.49 ± 7.91%
			625	(7.75 ± 5.54) × 10^6^	3.40 ± 0.64	99.92 ± 0.05%

**Table 4:** Binding pocket prediction and analysis of conserved germination protein via CASTp. The predicted binding pocket includes the pocket area, volume, and residues lining the pocket of conserved germination genes.

Bacteria	Protein	Pocket Area (Å^2^)	Volume (Å^3^)	Residue Lining Pocket
*B. cereus*	GPR	616.45	290.23	Arg196, Ser197, Ile198, Thr252, Ile253, Asp254, Phe255, Ile256, Leu257, Lys258, Phe260, Gly261, Arg262, Met264, Lys265, Ala304, Gly306, Glu309, Leu316, Leu319, Val320, Leu321, Ser322, Val330
	Lgt (GerF)	1284.21	1087.71	Trp3, Ile4, Val5, Arg6, Gln8, Pro9, Ser11, Leu12, Ile13, Gly15, Ser16, Gly19, Met23, Val41, Ala44, Phe45, 1le48, Ala49, Trp52, Lys53, Ile75, Lys79, His80, Val82, Cys85, Ile86, Ser89, Val90. Ile92, Leu107, Leu110, Pro111, Ile112, Ala113, Leu114, Cys115, Met116, Ser117, Ile118, Ile119, Phe120, Tyr 121, Glu154, Ala158, Leu159, Val162, Gly163, Leu165, Trp166, Ile178, Phe181, Leu182, Glu185, Gly186, His189, Phe203, Gly204, Met207, Gln208, Leu211, Ser212, Cys214, Val215, Leu218
*B. subtilis*	GPR	433.40	225.27	Arg44, His46, Lys50, Thr53, Asp55, Val56, Thr57, Glu59, Leu74, Ala76, Gln77, Gly78, Val90, Val93, Phe94, Glu96, Glu97, Ser99, Ala100, Phe101, Glu103, Asn104, Lys109, Ile206, Ile208, His355, Lys357, Val58, Ser359, Gln360, Asn362 Lys363, Gly364, Ser365, Tyr366,Asn367
	Lgt (GerF)	1565.20	1484.55	Leu18, Ala 19, His21, Tyr23, Gly24, Ile26, Ile27, Gly30, Ala31, Gly34, Ile37, Ala38, Arg40, Glu41, Lys44, Arg45, Gly46, Leu47, Phe52, Val56, Ala59, Ile60, Ala63, Ile64, Ala67, Ile89, Trp90, Gly93, Ile94, Gly99, Leu100, Ala103, Ile104, Thr106, Gly107, Leu116, Phe118, Lys120, Leu121, Ala122, Asp123, Ile124, Ala125, Ala126, Pro127, Ser128, Ile129, Leu 131, Gly132, Gln 133, Ile135, Gly136, Arg137, Gly139, Asn140, Glu145, Phe180, Glu183, Ser187, Ile192, Leu196, Arg198, Arg199, Ala200, Asn201, Leu202, Arg203, Arg204, Glu206, Met207, Phe208, Tyr211, Ile212, Tyr215, Arg219, Arg226, Thr233, Asp234, Ser235, Leu236

**Table 5:** Docking analysis of conserved germination proteins. The molecular interaction of TFDG with various genes. The table shows the best binding probability.

Bacteria	Protein	Docking Score (kcal/mol)	Interacting Residue	Distance	Category
*B. cereus*	GPR	−9.7	Trp120	2.51	Conventional H-bond
			Trp120	3.98, 3.88	Pi-Pi Stacked
			Asn121	2.59	Conventional H-bond
			Val163	2.78	Conventional H-bond
			Glu340	2.37	Conventional H-bond
	Lgt (GerF)	−7.6	Lys26	1.85	Conventional H-bond
			Lys26	3.73	Pi-Cation
			Ser31	2.99	Conventional H-bond
			Lys168	2.00	Conventional H-bond
			Arg171	2.51	Conventional H-bond
*B. subtilis*	GPR	−10.3	Gly119	2.06, 2.94	Conventional H-bond
			ASN120	2.34	Conventional H-bond
			ASN120	2.92	Conventional H-bond
			ASN120	2.03	Conventional H-bond
			ASN120	1.97	Conventional H-bond
			ASN122	1.68	Unfavorable Donor-Donor
			ASP126	2.97	Conventional H-bond
			Ile170	1.93	Conventional H-bond
			Thr172	3.05	Pi Donor H-bond
			Lys222	1.22	Unfavorable Donor-Donor
			Asp336	2.49	Conventional H-bond
			Asp336	2.23	Conventional H-bond
	Lgt (GerF)	−10.3	Ala63	3.78	Hydrophobic Pi-Sigma
			Ala103	3.62	Hydrophobic Pi-Sigma
			Ala103	2.07	Conventional H-Bond
			Ile129	3.29	Carbon Hydrogen Bond
			Gly132	3.96	Hydrophobic Pi-Sigma
			Tyr215	1.92	Conventional H-bond
			Asp234	2.38	Conventional H-bond

**Table 6:** Genes and protein information of conserved germination genes of *B. cereus* and *B. subtilis*.

Bacteria	Gene	NCBI Gene ID	Crystal Structure	PDB Reference	% Sequence Identity
*B. cereus*	*gpr*	56320307	None	IC8B	71.47%
	*lgt* (*gerF*)	56322894	None	5AZC	16.74%
*B. subtilis*	*gpr*	937838	None	IC8B	68.68%
	*lgt* (*gerF*)	12085459	None	5AZC	35.21%

**Table 7:** Grid box information of conserved germination protein for binding analysis.

Bacteria	Protein	Center X	Center Y	Center Z	Size X	Size Y	Size Z
*B. cereus*	GPR	−2	10	110	126	126	126
	Lgt (GerF)	−20	0	0	92	120	104
*B. subtilis*	GPR	−20	40	50	126	126	126
	Lgt (GerF)	−15	0	−8	90	114	126

## Data Availability

Data are contained within the article.

## Supplementary Materials

The following supporting information can be downloaded at: https://www.mdpi.com/article/10.3390/ijms23042153/s1.
