# Supplementary material for: Antibacterial and Sporicidal Activity Evaluation of Theaflavin-3,3′-digallate
Source: Int J Mol Sci. 2022 Feb 15;23(4):2153. doi: 10.3390/ijms23042153 (PMC8877948; doi:10.3390/ijms23042153)
Supplement: Supplementary file 1 [file ijms-23-02153-s001.zip › ijms-1522489-supplementary.pdf]

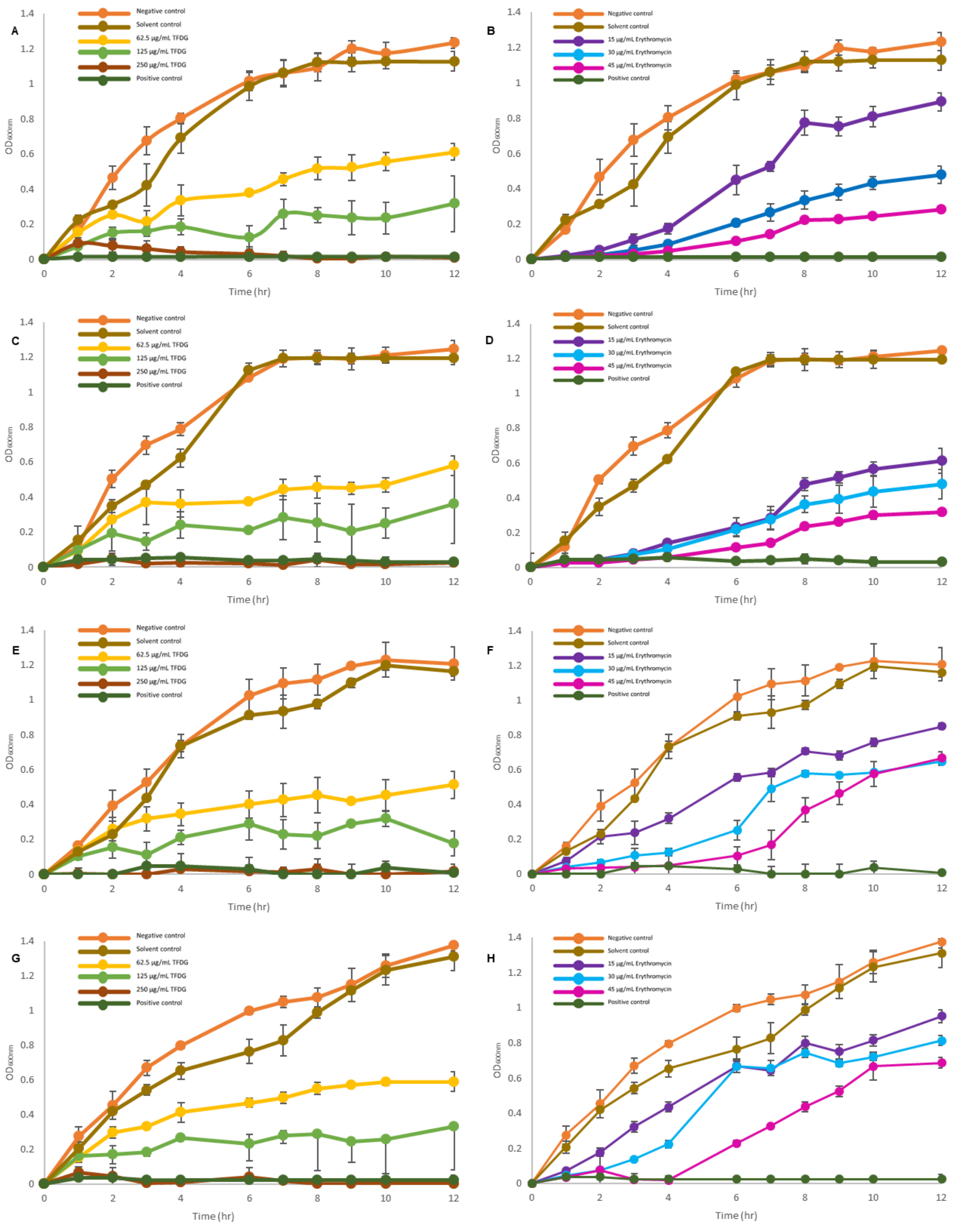

Figure S1. Growth analysis of Gram-negative species with various concentrations of TFDG and erythromycin. The orange is negative control (broth), light brown is solvent control (1% EtOH), yellow is 62.5 µg/mL TFDG, light green is 125 µg/mL TFDG, dark brown is 250 µg/mL TFDG, dark green is positive control (10% bleach), purple is 15 µg/mL erythromycin, light blue is 45 µg/mL erythromycin and pink is 45 µg/mL erythromycin **(A-B)** *K. aerogenes*, **(C-D)** *E. coli*, **(E-F)** *P. aeruginosa*, **(G-H)** *P. mirabilis*.

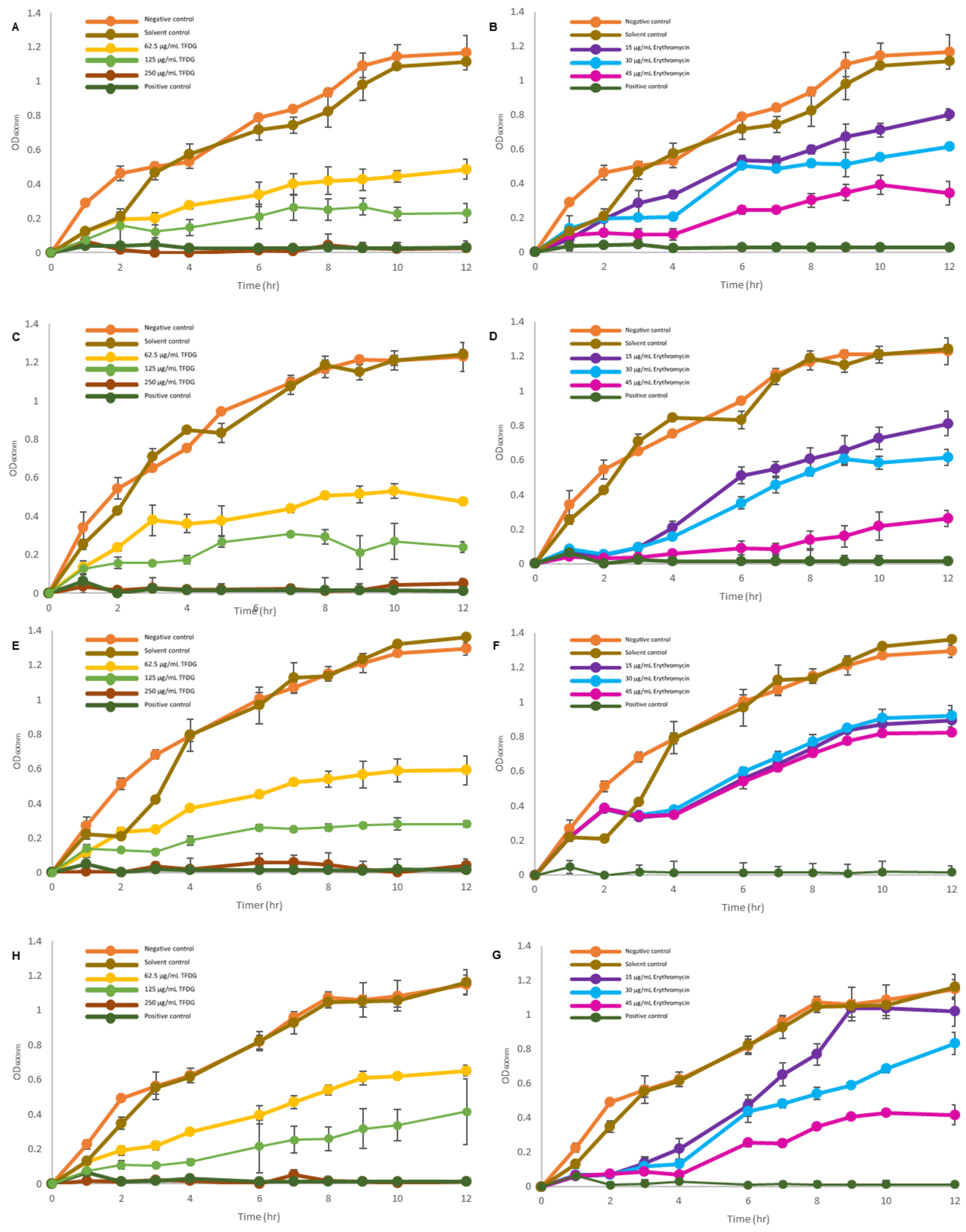

Figure S2. Growth analysis of Gram-positive species with various concentrations of TFDG and erythromycin. The orange is negative control (broth), light brown is solvent control (1% EtOH), yellow is 62.5 µg/mL TFDG, light green is 125 µg/mL TFDG, dark brown is 250 µg/mL TFDG, dark green is positive control (10% bleach), purple is 15 µg/mL erythromycin, light blue is 45 µg/mL erythromycin and pink is 45 µg/mL erythromycin **(A-B)** *B. cereus*, **(C-D)** *B. subtilis*, **(E-F)** *S. aureus*, **(G-H)** *S. pyogenes*.

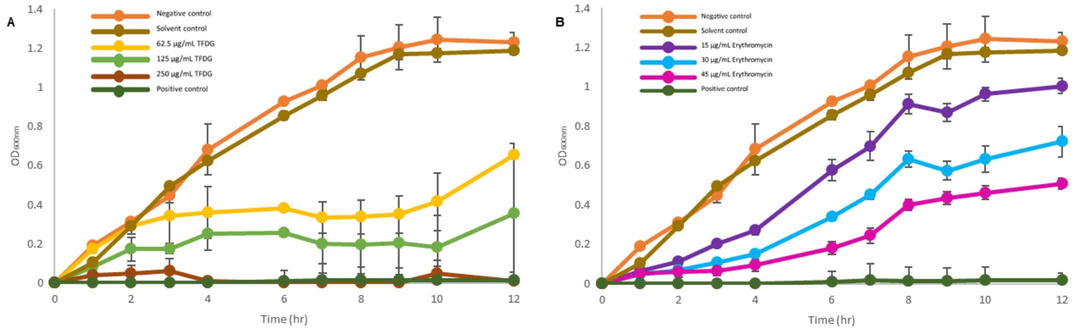

Figure S3. Growth analysis of acid-fast species with various concentrations of TFDG and erythromycin. The orange is negative control (broth), light brown is solvent control (1% EtOH), yellow is 62.5 μg/mL TFDG, light green is 125 μg/mL TFDG, dark brown is 250 μg/mL TFDG, dark green is positive control (10% bleach), purple is 15 μg/mL erythromycin, light blue is 45 μg/mL erythromycin and pink is 45 μg/mL erythromycin **(A-B)** *M. smegmatis*.
